# Machine-learning facilitates selection of a novel diagnostic panel of metabolites for the detection of heart failure

**DOI:** 10.1038/s41598-019-56889-8

**Published:** 2020-01-10

**Authors:** M. Marcinkiewicz-Siemion, M. Kaminski, M. Ciborowski, K. Ptaszynska-Kopczynska, A. Szpakowicz, A. Lisowska, M. Jasiewicz, E. Tarasiuk, A. Kretowski, B. Sobkowicz, K. A. Kaminski

**Affiliations:** 1 Medical University of Bialystok, Cardiology Department, M. Sklodowskiej-Curie 24A, 15-276 Bialystok, Poland; 2 Medical University of Bialystok, Clinical Research Centre, M. Sklodowskiej-Curie 24A, 15-276 Bialystok, Poland; 3 Department of Population Medicine and Civilization Diseases Prevention, Medical University of Bialystok, Waszyngtona 13A, 15-276 Bialystok, Poland

**Keywords:** Heart failure, Heart failure

## Abstract

The metabolic derangement is common in heart failure with reduced ejection fraction (HFrEF). The aim of the study was to check feasibility of the combined approach of untargeted metabolomics and machine learning to create a simple and potentially clinically useful diagnostic panel for HFrEF. The study included 67 chronic HFrEF patients (left ventricular ejection fraction-LVEF 24.3 ± 5.9%) and 39 controls without the disease. Fasting serum samples were fingerprinted by liquid chromatography-mass spectrometry. Feature selection based on random-forest models fitted to resampled data and followed by linear modelling, resulted in selection of eight metabolites (uric acid, two isomers of LPC 18:2, LPC 20:1, deoxycholic acid, docosahexaenoic acid and one unknown metabolite), demonstrating their predictive value in HFrEF. The accuracy of a model based on metabolites panel was comparable to BNP (0.85 vs 0.82), as verified on the test set. Selected metabolites correlated with clinical, echocardiographic and functional parameters. The combination of two innovative tools (metabolomics and machine-learning methods), both unrestrained by the gaps in the current knowledge, enables identification of a novel diagnostic panel. Its diagnostic value seems to be comparable to BNP. Large scale, multi-center studies using validated targeted methods are crucial to confirm clinical utility of proposed markers.

## Introduction

Despite significant improvement in pharmacological and invasive treatment of cardiovascular diseases (CVD) that led to an increase in life expectancy, the incidence of heart failure with reduced ejection fraction (HFrEF) continues to rise. As a result, HFrEF has become a major public health problem as it constitutes currently a leading cause of hospital admissions over the age of 65^1^. In spite of rapidly expanding current knowledge, molecular basis of the HFrEF is still incompletely understood. Available biomarkers, though playing an important role in the HFrEF diagnosis, risk stratification and prognosis (eg.: B-type natriuretic peptide – BNP, N-terminal pro B-type natriuretic peptide - NTproBNP, suppression of tumorigenicity-2 - ST2 family, galectin-3 - Gal-3, troponin - Tn etc.), do not give a satisfactory answer about HFrEF pathophysiology. Moreover there are some significant limitations that may lower clinical utility of natriuretic peptides (NPs: BNP or NTproBNP) which are most established HF biomarkers in clinical practice (e.g. limitation in detection of early and asymptomatic stages of HFrEF, an increase in NPs concentration due to the several cardiac as well as non-cardiac conditions, NPs concentration rising with age, are higher in women, while lower in obese patients). None of currently available HF biomarker do provide additional therapeutic target possibilities nor enable biomarker guided therapy in chronic HFrEF^2^. In fact, an assessment of a single, particular metabolite or protein cannot provide fully informative results. Each of the currently available HFrEF biomarkers being restricted to particular mechanism (e.g. NPs – pressure-volume overload, ST2 family – inflammation, Gal-3 – fibrosis and cardiac remodeling etc.) cannot reflect simultaneously all other mechanisms related to HFrEF pathophysiology. HFrEF being a multisystemic syndrome^3^ needs a holistic approach to study systemic changes occurring in the course of HFrEF development. Due to the aforementioned limitations there is still a need to search of newer HFrEF biomarkers with particular emphasis on a multibiomarker approach. Untargeted metabolomics analysis enables comprehensive characterization of low molecular weight metabolites reflecting the complete metabolic phenotype of the disease. Therefore the importance of this hypothesis free, complex metabolic evaluation has increased within the last years and metabolomics approach has been more frequently used in the search for new HFrEF biomarkers. However, the number of small-molecule metabolites detected by using untargeted metabolomics approach range from hundreds to thousands. As a result, a problem of massive amounts of data has appeared generating a need of specialized forms of data analysis. Studies that have been conducted so far were restricted mainly to classical regression-based models which constitute significant limitation especially in terms of pre-specification of a model structure (based on a theory and assumption), the number of variables included in the analysis and their interactions. To our knowledge, in none of the available HFrEF untargeted metabolomics studies machine-learning (ML) algorithms have been used to analyze metabolomics data. ML, by having different motivating philosophies (data-driven models) and by not being limited by current knowledge (no need for a pre-specification of a model structure), seems to be a powerful tool to improve diagnostic and prognostic processes in various diseases^4–7^. Therefore, the aim of the study was to detect in peripheral blood possibly all low molecular weight metabolites which differentiate HFrEF patients from controls with further creation of the top performing diagnostic panel using ML algorithms.

## Material and Methods

### Study population

Study population, methodology for obtaining peripheral blood samples, clinical and metabolomics evaluation were as described previously^8^. In brief, 67 patients with chronic heart failure with reduced ejection fraction (HFrEF) and 39 age-, ischemic heart disease (IHD) occurrence- and body mass index (BMI)-matched controls were enrolled in the study. Patients’ (HFrEF and control group) inclusion to the study was conducted between 2012–2015 at Cardiology Department of the University Hospital in Bialystok, Poland. The investigation conforms with the principles outlined in the Declaration of Helsinki. The Bioethical Commission of the Medical University of Bialystok approved the research (R-I-002/67/2013). HFrEF study group consisted of ambulatory optimally treated patients (according to the 2012 European Society of Cardiology guidelines for the diagnosis and treatment of acute and chronic heart failure) with stable moderate chronic HFrEF (left ventricular ejection fraction – LVEF ≤ 35%) who did not have an episode of decompensation within the last month. All of the HFrEF patients had a minimum six-month history of the disease. Clinical, biochemical (BNP) and echocardiographic (LVEF ≥ 50%) assessment allowed to exclude HF from the control group which consisted of ambulatory treated patients with arterial hypertension, atrial fibrillation, ischemic heart disease and/or hypercholesterolemia. All of the study participants provided written informed consent. The exclusion criteria were the same for both groups: acute and chronic inflammatory diseases (rheumatoid arthritis and asthma), severe chronic obstructive pulmonary disease (forced expiratory volume in one second - FEV1 less than 50% of a predicted value), severe renal dysfunction (estimated glomerular filtration rate – eGFR < 30 ml/min/1.73 m²), diabetes mellitus, thyroid dysfunction requiring pharmacotherapy, a history of implantation of the cardiac resynchronization therapy device (CRT) or diagnosis of cancer in the past five years. Information about treatment, prior hospitalizations and concomitant diseases were gathered from the medical documentation. Clinical (body mass index - BMI, systolic and diastolic blood pressure – SBP/DBP, heart rate - HR, New York Heart Association - NYHA class), biochemical (complete blood count, iron level, parameters of renal function, urea, uric acid, fasting lipid profile, natriuretic peptide – BNP, C reactive protein – CRP), echocardiographic (left ventricular ejection fraction – LVEF, left ventricular end-diastolic diameter – LVEDd) and functional (six-minute walk test – 6MWT, cardiopulmonary exercise test – CPET with rest spirometry) assessment were carried out in all of the enrolled patients. Clinical examination, basic biochemical analyses, echocardiography and functional assessment were done at the day of inclusion to the study. Metabolomics assessment was performed in two batches in 2014 and 2015 after collecting complete sets of biological material within the HFrEF and control group. Each batch included both HFrEF and control group patients.

### Clinical parameters

BMI was calculated as weight [kg]/height [m]². The modification of diet in renal disease (MDRD) formula GFR calculator was used to compute estimated glomerular filtration rate (eGFR). The incidence of ischaemic heart disease (IHD) was calculated based on medical documentation (HFrEF - invasively confirmed IHD as a cause of chronic HF; control group - previous diagnosis of IHD). For cardiopulmonary exercise test (CPET), a standardized continuous Ball State University/Bruce (BSU/Bruce) Ramp treadmill protocol was used with 20-second stages and continuous increase in workload per stage. Forty four (66%) patients with chronic heart failure carried out CPET. The decision about patient’s ability to perform the CPET was made after the 6MWT. Difficulties with walking due to non-cardiac causes (e.g. peripheral occlusive arterial disease, problems with lumbar spine), significant fatigue in the 6MWT, fear of exercise on a treadmill or mask intolerance were main reasons of not performing CPET.

Peak oxygen uptake (PVO2) is the rate of an oxygen consumption reflecting the difference between inspired and expired volume of oxygen. PVO2 is measured at peak exercise on a treadmill and expressed as milliliters of O_2_ per kg per minute (mL/kg/min). PVO2 depends on the arteriovenous O_2_ difference and cardiac output reserve. The slope of a minute ventilation to carbon dioxide output (VE/VCO2 slope) reflects the effectiveness of ventilation. An increase in VE/VCO2 slope in HFrEF patients is known as an indicator of poor outcome.

### Metabolic fingerprinting by LC-QTOF-MS

In order to avoid variation due to circadian rhythm, the collection of peripheral venous blood samples was carried out in the morning (at the day of inclusion to the study) between 8.00 and 10.00 am after compulsory overnight fasting (at least 8 h). Samples were further centrifuged for 10 minutes (1300 x g, room temperature). The separated serum was stored in the Eppendorf tubes at −80 °C until further metabolomics analysis. All of the morning medications were taken as usual. Collected serum samples were further subjected to untargeted analysis by liquid chromatography - quadrupole time-of-flight - mass spectrometry (LC-QTOF-MS - model 6550, Agilent Technologies, Santa Clara, California, USA) system. The analytical process was controlled by the use of quality control (QC) samples^9^. As the LC-MS analyses were performed in two separate sets (1^st^ set: 36 HFrEF patients and 19 age-matched controls; 2^nd^ set: 31 HFrEF patients and 20 age-, gender- and concomitant disease-matched controls), a pool of equal volumes of serum from each of the 55 samples in derivation and 51 in validation sets were used to prepare the QCs. They were prepared independently following the same procedure as for the rest of the samples and injected at the beginning of the run and after every 6–7 real samples. Samples were analyzed by the HPLC system that consisted of a degasser, two binary pumps and thermostated auto sampler connected to a mass spectrometry detector using previously described method^8^. Samples from each set (derivation and validation) were analyzed in a randomized order in separate runs (first for positive and then for negative ion mode). Metabolomics analyses were conducted at Clinical Research Centre of Medical University of Bialystok, Poland.

Processing of LC-MS data was performed as described previously^8^. Briefly, the raw data collected by the analytical instrumentation was cleaned of background noise and unrelated ions by the Molecular Feature Extraction (MFE) tool in the Mass Hunter Qualitative Analysis Software (B.06.00, Agilent). Alignment, quality assurance^9^ and data filtering were performed using Mass Profiler Professional (MPP) 12.6.1 (Agilent).

### Statistical analysis

Basic statistical analyses were performed as described previously^8^. In short, continuous variables are expressed as the mean ± standard deviation (SD) or median and interquartile range (IQR), depending on the type of distribution. Categorical variables are presented as raw values and percentages from the total. The Student’s t-test, Mann-Whitney U test or χ² test were used, as appropriate. Statistical significance was defined as p < 0.05.

As the data were obtained by subjecting samples to MS analysis in two separate batches, which imposed further manual matching, the variables were prefiltered to include 50% most variant metabolites of these present in at least 80% of samples. Following the filtering, manual matching and elimination of artifacts (signals present in blank analysis), 63 variables from both ESI ion modes have been subjected to computational modelling. Missing values were replaced by k-means nearest neighbour analysis according to the criteria of Armitage *et al*.^10^.

There were nine cases with missing BNP, which were omitted in models. No data imputation techniques were applied, since gathered data would not allow us to obtain a reliable approximation of unobserved BNP levels. Within each batch, the variables were transformed with Yeo-Johnson method and standardized by subtracting the mean and dividing by trimmed standard deviation. Cases were split into training and test set, with proportions of 3:2. Recursive feature elimination with repeated cross-validation (RFECV) based on random forests was performed on the training set to rank the predictors. Top 20 ranking metabolites were then subset and incrementally used as predictors of multiple GLMs fitted to training set, with their accuracy assessed on resampling. The top performing model, containing 8 predictors, was chosen and its validity was assessed on the test set. Additional multiple regression models, including HFrEF presence and another clinical covariate as predictors, one for each clinical covariate and metabolite combination, have been built to assess potential confounding on metabolite levels. Correlations between serum intensities of metabolites included in the panel and clinical parameters were performed calculating Pearson’s product-moment coefficients for pairs of normalized variables. The analysis was performed in R version 3.4.2.

### Metabolites identification

Identification of significant metabolites was performed as described previously^8^. Identification of uric acid, deoxycholic acid and docosahexaenoic acid was confirmed by matching retention time, accurate mass and fragmentation pattern of authentic standards (Sigma Aldrich). Lysophospholipids were identified based on previously described fragmentation pattern^9^.

### Equation 1

Model equation, for the logit-link binomial model, relating HFrEF occurrence (outcome) to metabolite panel (predictors). Predictors are 0-centered, Yeo-Johnson normalized intensities of metabolites.

$$p=\frac{1}{1+{{\rm{e}}}^{-y}}$$

y = 1.781 + −3.190 * [LPC 18:2sn2] + 0.851 * [UA] + 0.037 * [LPC 18:2sn1] + −0.035 * [UM] + 2.749 * [LPC 18:2sn2] + −1.099 * [LPC 20:1] + −0.673 * [DA] + −1.099 * [DHA].

## Results

### Clinical group characteristics

The basic clinical characteristics of study groups (HFrEF and controls) is shown in Table 1. There were no statistically significant differences between study groups in terms of age, blood pressure, BMI and IHD occurrence. The percent of women was higher within the control group (n = 11, 29% vs n = 7, 10%, respectively; p = 0.019). HFrEF group consisted of mildly/moderately symptomatic patients (NYHA class II – 43%; III − 57%) who had significantly impaired left ventricular ejection fraction (EF 24.3 ± 5.9%). Ischemic heart disease was an etiology of HFrEF in 57% of patients. HFrEF patients presented slower resting heart rate than controls (70.7 ± 9.9 vs 75.3 ± 12.5 beats per minute, p = 0.045). Among assessed laboratory parameters higher concentration of uric acid (6.8 mg/dL IQR 6.0–7.9 mg/dL vs 5.99 mg/dL IQR 5.1–7.0 mg/dL, p = 0.029) and lower total cholesterol (167.5 ± 42 mmo/L vs 197.2 ± 35 mg/dL, p = 0.001), HDL (46.5 ± 14.1 mmol/L vs 53.3 ± 14.3 mmol/L, p = 0.029), LDL (101.9 ± 32.7 mmol/L vs 127.0 ± 39.2 mmol/L, p = 0.001) levels were observed in HFrEF patients. Pharmacological treatment of HFrEF (ACEIs/ARBs, B-blockers, MRAs) was consistent with the current ESC recommendations. HFrEF patients were more frequently treated with the use of statins, ASA, ACEIs, B-blockers and MRAs in comparison with those without the disease. Whereas CCBs were used more often in the control group.

**Table 1:** Basic clinical characteristics of patients included in the study (chronic heart failure and controls without the disease).

	Chronic heart failure (HFrEF, n = 67)	Controls (n = 39)	P-value
Age [years]²	62.6 (12.3)	62.6 (10.4)	0.997
Male gender [n, %]	60 (90)	28 (72)	**0.019**
NYHA class II/III [n, %]	29 (43)/38 (57)	—	—
SBP [mmHg]²	127.8 (23.7)	133.7 (18.4)	0.197
DBP [mmHg]²	77.7 (14.5)	79.3 (7.2)	0.521
HR [bpm]²	70.7 (9.9)	75.3 (12.5)	**0.045**
BMI [kg/m²]²	28.6 (4.1)	28.1 (4.7)	0.602
**Laboratory results**			
RBC [mln/mm³]²	4.7 (0.5)	4.8 (0.5)	0.174
Hb [g/dl]²	14.2 (1.2)	14.2 (1.9)	0.808
Fe [μg/dL]²	111.3 (57.9)	106.0 (40.1)	0.641
CRP [mg/dL]²	1.8 (0.9–3.0)	1.6 (1.0–2.5)	0.702
eGFR [ml/min/1.73 m²]²	73.2 (19.6)	80.3 (15.7)	0.057
Uric acid [mg/dL]²	6.8 (6.0–7.9)	5.99 (5.1–7.0)	**0.029**
Urea [mg/dL]²	45.8 (16.1)	40.6 (19.4)	0.239
TChol [mmol/L]²	167.5 (42.0)	197.2 (35.0)	**0.001**
LDL [mmol/L]²	101.9 (32.7)	127.0 (39.2)	**0.001**
HDL [mmol/L]²	46.5 (14.1)	53.3 (14.3)	**0.029**
TG [mmol/L]²	118.1 (68.6)	124.9 (57.1)	0.626
BNP [pg/mL]¹	183.8 (89–279)	24.3 (13.7–54.0)	**<0.0001**
**Echocardiography**			
LVEF [%]²	24.3 (5.9)	61.2 (5.1)	**<0.0001**
LVEDd [mm]²	6.7 (1.1)	4.9 (0.5)	**<0.0001**
**Functional capacity**			
6MWT [m]²	382.1 (118.1)	488.5 (128.2)	**<0.0001**
CPET duration [min]¹	6 (4–9)*	11 (8–12)	**0.001**
Peak VO2 [ml/kg/min]²	17.2 (6.6)	23.3 (5.5)	**<0.0001**
VE/VCO2 slope²	31.6 (8.7)	26.8 (3.6)	**0.008**
**Comorbidities**			
IHD [n, %]	38 (57)^∫^	18 (46)**	0.294
AF [n, %]	19 (28)	6 (15)	0.129
CKD [n, %]	54 (81)	32 (80)	0.980
**Medications**			
Statin use [n,%]	53 (79)	20 (51)	**0.003**
ASA [n,%]	43 (64)	13 (33)	**0.002**
ACEIs/ARBs [n,%]	63 (94)	25 (64)	**<0.0001**
Β-blockers [n,%]	64 (95)	20 (51)	**<0.0001**
MRAs [n,%]	65 (97)	2 (5)	**<0.0001**
Thiazide diuretics [n,%]	4 (6)	3 (8)	0.731
CCBs [n,%]	6 (9)	10 (26)	**0.021**
Allopurinol [n,%]	11 (16)	3 (8)	0.201

¹Median (IQR); ²Mean (SD); IQR – interquartile range; SD – standard deviation;

*CPET was performed in 66% (n = 44) of HFrEF patients; ^∫^IHD – invasively confirmed ischaemic heart disease as the etiology of heart failure; **IHD – invasively confirmed or clinically diagnosed and pharmacologically treated ischaemic heart disease;

HFrEF – heart failure with reduced ejection fraction; NYHA class – New York Heart Association functional classification; SBP/DBP – systolic/diastolic blood pressure; MAP – mean arterial pressure; HR – heart rate; BMI – body mass index; RBC – red blood cells; Hb – haemoglobin; Fe – serum iron level; CRP – C-reactive protein; eGFR – estimated glomerular filtration rate; TChol – total cholesterol; LDL – low-density lipoproteins; HDL – high-density lipoproteins; TG – triglycerides; BNP – B-type natriuretic peptide; LVEF – left ventricular ejection fraction; LVEDd – left ventricle end-diastolic diameter; 6MWT – six-minute walk test; CPET duration – cardiopulmonary exercise test duration; Peak VO2 – peak rate of oxygen uptake; VE/VCO2 slope – minute ventilation/carbon dioxide production slope; AF – atrial fibrillation; CKD – chronic kidney disease (eGFR <90 ml/min/1.73 m²); ASA – acetylsalicylic acid; ACEIs/ARBs – angiotensin-converting-enzyme inhibitors/angiotensin II receptor blockers; MRAs – mineralocorticoid receptor antagonists; CCBs – calcium channel blockers.

### Selection of top ranked metabolites which created the diagnostic panel

**Figure 1:**
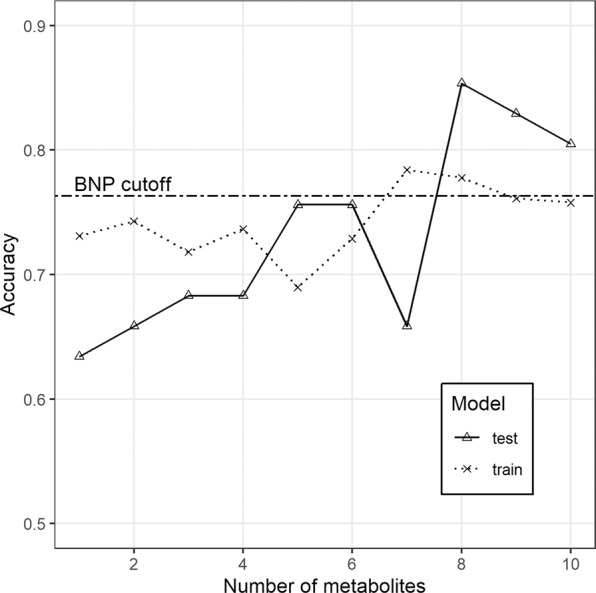
Accuracy (%) for metabolites’ panel (in relation to the number of predictors included in the panel) in comparison with the accuracy for BNP while assuming a cut-off point equal 35 pg/dl (as for the diagnosis of heart failure of non-acute onset).

**Figure 2:**
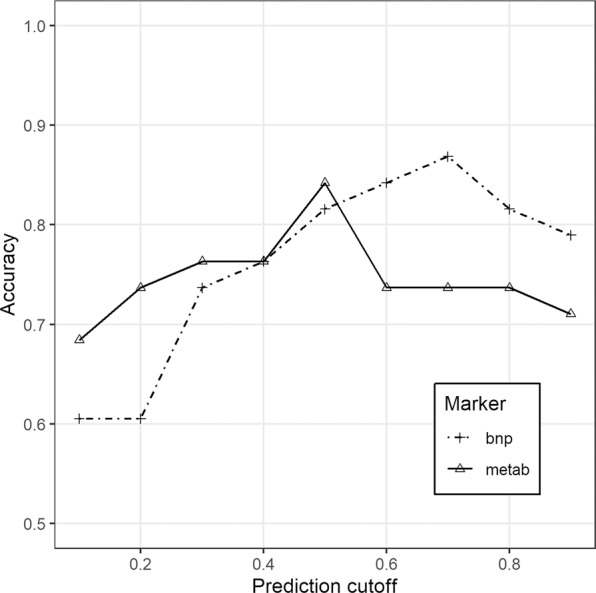
Accuracy (%) for metabolites’ panel in relation to the single cut-off allowing discrimination of HFrEF cases.

**Figure 3:**
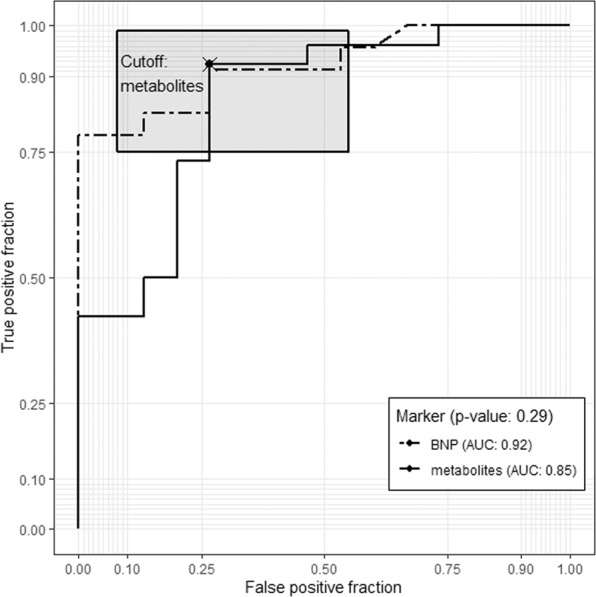
Receiver operating curve (ROC) for eight metabolites’ panel and BNP (sensitivity 0.73 vs 0.80, specificity 0.92 vs 0.83; AUC 0.85 vs 0.92, respectively; p = 0.29).

**Figure 4:**
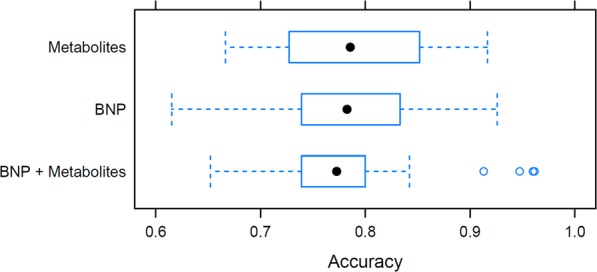
Comparison of model accuracies estimated on 25 runs of data resampling.

Quality assurance protocol, manual matching and filtering of further artifacts were carried out on the data obtained from the two independent LC-QTOF-MS analyses. As a result, 63 variables have been subjected to computational modelling. Random forests nested inside RFECV algorithm lead to selection of 20 top ranked predictors, further sequentially used as covariates of GLMs, of which the most accurate final model was chosen, built on 8 metabolites (Fig. 1, Table 2). Further MS/MS spectrum analyses enabled to identify components of the model (uric acid, two isomers of lysophosphatidylcholine - LPC 18:2, LPC 20:1, deoxycholic acid, docosahexaenoic acid and one unknown metabolite), and build the final model equation (Equation 1). Apart from uric acid serum intensity of seven remaining metabolites was significantly lower in HFrEF (Table 3). Based on both accuracy profiling (Fig. 2), and ROC curve (Fig. 3), a prediction cut-off value equal to 0.5 was found to be optimally discriminate between controls and HFrEF cases. Our model compared to BNP as a sole predictor presents with better accuracy (0.85 vs 0.82; Fig. 1) and specificity (0.92 vs 0.83), but worse sensitivity (0.73 vs 0.80). Overall performance measured by AUC is insignificantly lower (0.85 vs 0.92, p = 0.29). To demonstrate the additive value of metabolite panel on top of BNP, we have built an additional model containing all these predictors (Supplementary Table 1). Formal testing confirmed better goodness-of-fit of full model than BNP alone (p = 0.001), while resampling profile indicates comparable accuracy (Fig. 4). Additional multiple linear regression models have been built, to investigate the potential effect of other clinically relevant covariates (such as ischaemic ethiology, statin or ACEI treatment) on metabolite levels (Supplementary Tables 2–4). Although in some cases these have shown some extent of potential confounding, they did not nullify the initial relationship with HFrEF.

**Table 2:** Estimates for parameters of the proposed model.

Model Term	Coef. Estimate	Coef. Std. Error	P-value
(Intercept)	1.781	0.649	0.006
LPC 18:2sn2	−3.19	2.478	0.198
UA	0.851	0.391	0.029
LPC 18:2sn1	0.037	0.771	0.962
UM	−0.035	0.87	0.967
LPC 18:2sn2	2.749	2.691	0.307
LPC 20:1	−1.099	0.399	0.006
DA	−0.673	0.33	0.042
DHA	−1.099	0.414	0.008

P-value for the model (LRT vs null model): 2.369 * 10^−08^.

**Table 3:** Mean serum intensities of top ranked metabolites included in the panel in chronic heart failure versus control group (without partitioning into training and test set).

Metabolite	RT [min]	m/z	Monoisotopic mass [Da]	Adduct	HFrEF (n = 67)	±SD	CONTROL GROUP (n = 39)	±SD	P-value
UA	0.3	169.036	168.028	[M + H^+^]^+^	0.294	1.50	−0.664	1.26	0.001
LPC 18:2sn2	5.5	565.339	519.333	[M+HCOO^−^]^−^	−0.359	1.34	0.671	1.42	<0.0001
LPC 18:2sn1	5.4	565.339	519.333	[M + HCOO^−^]^−^	−0.441	1.73	0.374	1.64	0.018
UM	5.5	700.305	701.313	[M−H^+^]^+^	−0.347	1.38	0.476	1.54	0.007
LPC 18:2sn2	5.5	504.311	519.333	[M + HCOO−HCOOCH_3_]^−^	−0.337	1.37	0.599	1.45	0.002
LPC 20:1	5.7	676.306	549.379	[M+TFA − TFA−CH_3_]^−^	−0.701	2.55	0.785	1.16	<0.0001
DA	5.2	391.285	392.293	[M−H^+^]^+^	−0.519	1.80	0.677	1.84	0.002
DHA	7.2	329.248	328.240	[M + H^+^]^+^	−0.276	1.44	0.475	1.15	0.004

RT – retention time; m/z – mass-to-charge ratio; HFrEF – heart failure with reduced ejection fraction; SD – standard deviation; UA – uric acid; LPC – lysophosphatidylcholine; UM – unknown metabolite; DA – deoxycholic acid; DHA – docosahexaenoic acid.

Serum intensities of all metabolites significantly correlated with left ventricular ejection fraction. Moreover, apart from UA and DHA, serum intensities of remaining metabolites moderately positively correlated with exercise duration on a treadmill, peak oxygen consumption, renal function and negatively correlated with VE/VCO2 slope. Serum intensities of lysophosphatidylcholines (LPCs) included in the metabolites’ panel correlated positively with LDL and total cholesterol serum level. Weak negative correlation was also observed between deoxycholic acid, LPC 18:2 sn1 and BNP (Table 4).

**Table 4:** Correlations between serum intensities of 8 metabolites included in the panel and clinical parameters (all patients - HFrEF and control group, n = 106).

	DA		DHA		LPC 18:2 sn1		LPC 18:2 sn2*		LPC 18:2 sn2**		LPC 20:1		UA		UM	
	r	p-value	r	p-value	r	p-value	r	p-value	r	p-value	r	p-value	r	p-value	r	p-value
Age [years]	—	NS	—	NS	**−0.33**	**<0.0001**	**−0.26**	**0.006**	**−0.26**	**0.008**	**−0.28**	**0.004**	—	NS	**−0.22**	**0.024**
BMI [kg/m^2^]	**0.22**	**0.027**	—	NS	—	NS	**−0.29**	**0.003**	**−0.28**	**0.004**	—	NS	**0.20**	**0.045**	**−0.23**	**0.020**
BNP [pg/mL]	**−0.25**	**0.015**	—	NS	**−0.21**	**0.043**	—	NS	—	NS	—	NS	—	NS	—	NS
CRP [mg/dL]	—	NS	—	NS	—	NS	—	NS	**−0.21**	**0.037**	—	NS	**0.20**	**0.039**	—	NS
eGFR [ml/min/1.73 m^2^]	—	NS	—	NS	**0.30**	**0.002**	**0.30**	**0.002**	**0.30**	**0.002**	**0.21**	**0.029**	**−0.32**	**<0.0001**	**0.27**	**0.005**
Urea [mg/dL]	—	NS	—	NS	**−0.23**	**0.039**	—	NS	—	NS	—	NS	**0.29**	**0.007**	—	NS
TChol [mmol/L]	—	NS	—	NS	**0.32**	**0.001**	**0.41**	**<0.0001**	**0.42**	**<0.0001**	**0.26**	**0.008**	—	NS	**0.43**	**<0.0001**
LDL [mmol/L]	—	NS	—	NS	**0.37**	**<0.0001**	**0.37**	**<0.0001**	**0.39**	**<0.0001**	**0.29**	**0.004**	—	NS	**0.42**	**<0.0001**
HDL [mmol/L]	—	NS	—	NS	—	NS	**0.36**	**<0.0001**	**0.35**	**<0.0001**	—	NS	—	NS	**0.23**	**0.021**
UA [mg/dL]	—	NS	—	NS	—	NS	**−0.26**	**0.019**	**−0.28**	**0.011**	—	NS	**0.84**	**0.000**	**−0.24**	**0.034**
LVEF [%]	**0.34**	**<0.0001**	**0.29**	**0.003**	**0.22**	**0.026**	**0.31**	**0.002**	**0.34**	**<0.0001**	**0.33**	**<0.0001**	**−0.33**	**<0.0001**	**0.27**	**0.006**
LVEDd [mm]	**−0.23**	**0.028**	**−0.29**	**0.004**	—	NS	—	NS	—	NS	—	NS	**0.39**	**<0.0001**	—	NS
6MWT [m]	—	NS	—	NS	**0.25**	**0.022**	**0.36**	**<0.0001**	**0.37**	**<0.0001**	—	NS	—	NS	**0.25**	**0.019**
Ex. duration [min]	**0.25**	**0.033**	—	NS	**0.32**	**0.005**	**0.43**	**<0.0001**	**0.45**	**<0.0001**	**0.39**	**<0.0001**	—	NS	**0.42**	**<0.0001**
Peak VO2 [ml/kg/min]	**0.26**	**0.025**	—	NS	**0.34**	**0.003**	**0.49**	**<0.0001**	**0.51**	**<0.0001**	**0.29**	**0.014**	—	NS	**0.43**	**<0.0001**
Ve/VCO2 slope	**−0.31**	**0.008**	—	NS	**−0.24**	**0.042**	**−0.27**	**0.022**	**−0.27**	**0.022**	—	NS	—	NS	**−0.25**	**0.030**

^*^m/z = 504.311; **m/z = 565.339; DA – deoxycholic acid; DHA – docosahexaenoic acid; LPC – lysophosphatidylcholine; UA – uric acid; UM – unknown metabolite; BMI – body mass index; BNP – B-type natriuretic peptide; CRP – C-reactive protein; eGFR – estimated glomerular filtration rate; TChol – total cholesterol; LDL – low-density lipoproteins; HDL – high-density lipoproteins; UA – uric acid; LVEF – left ventricular ejection fraction; LVEDd – left ventricle end-diastolic diameter; 6MWT – six-minute walk test; Ex. duration – cardiopulmonary exercise test duration (CPET was performed in 66%, n = 44, of HFrEF patients); Peak VO2 – peak rate of oxygen uptake; VE/VCO2 slope – minute ventilation/carbon dioxide production slope.

## Discussion

As heart failure is not a single organ disease but a multisystemic syndrome, variety of systemic changes occur in the course of HFrEF development. Analysis of changes in blood metabolites profile seems to be an attractive approach to perform holistic assessment of complex adaptive responses and to discover valuable novel HFrEF biomarkers. An untargeted metabolomics analysis being unrestricted by current knowledge enables detection of possibly all low molecular weight metabolites present in a particular moment in the peripheral blood. That allows a simultaneous assessment of changes in many various metabolites, including even those that we have not been able to identify to date. Our study presents a completely new approach towards biomarkers – something to be expected in the era of personalized medicine when “one fits all” approach will be replaced by analysis of simultaneous changes of multiple substances reflecting different mechanisms. According to current research, collective biomarkers better reflect the complexity of changes in the organism and possibly will increase likelihood to define particular sub-phenotypes of diseases (in this case heart failure)^11–13^. Previous research has already proven that metabolomics may constitute a powerful diagnostic and prognostic tool in chronic HF^14,15^. However, a high diversity of analytical methods (e.g. nuclear magnetic resonance – NMR, mass spectrometry – MS), separation techniques (i.e. capillary electrophoresis or chromatography – gas or liquid) and approaches (untargeted, targeted) used to date to study metabolome as well as various studied populations are partly responsible for a diversity in the results obtained by different research groups. For instance, Cheng *et al*.^14^ have performed untargeted metabolomics analysis followed by targeted evaluation of obtained results and an identification of a combination of four metabolites (histidine, phenylalanine, spermidine, and phosphatidylcholine C34:4) that discriminated HF stage C from control group similarly to b-type natriuretic peptide (BNP). Authors have suggested that profile of metabolites (another panel identified by Cheng *et al*.) may provide even better prognostic value in comparison with conventional biomarkers such as BNP in HF patients. Mueller-Hennessen *et al*.^16^ have created a lipid diagnostic panel which improved HFrEF detection, even in mild and asymptomatic stages. Hunter *et al*.^17^ have indicated a group of circulating metabolites which were significantly elevated in HF in comparison with the control group. Moreover it allowed to differentiate HFrEF from heart failure with preserved ejection fraction (HFpEF). Different analytical approaches were used in all of those studies (Cheng *et al*. – untargeted followed by targeted analysis; Mueller-Hennessen *et al*. – metabolite profiling; Hunter *et al*. – targeted analysis). Apart from this, analyses carried out by Cheng *et al*.^14^ and Hunter *et al*.^17^ included healthy control group. As the metabolites’ profile is susceptible to many various external (e.g. pharmacotherapy) and internal (e.g. age, sex, comorbidities, renal function) factors, many differences in basic characteristic of HF and control group (e.g. age, BMI, comorbidities, renal function, pharmacotherapy) observed in those studies may serve as significant confounding factors. On the contrary to previous research, control group in our study was carefully matched in terms of age, BMI, eGFR, IHD occurrence. Despite this, differences in medication were noticed. Additional analyses showed the relation between medications and serum intensities of UA (ACEI treatment), LPC 18:2sn1 (ACEI, statin therapy) and HFrEF (Supplementary Tables 3, 4). However, due to small group size these analyses are biased by strong association between the treatment and the presence of heart failure. Previous metabolomics studies^14^ that used untargeted analysis to select metabolites and further create a novel diagnostic panel for HFrEF have been based on classical, hypothetical-driven statistical approaches. In fact, the amount of data gathered from an untargeted metabolomics analyses poses an analytical challenge resulting from a necessity of modelling a multidimensional space of relations between metabolite fingerprint and outcomes, and simplifying the results just enough to facilitate holistic, straightforward conclusions. An application of machine-learning techniques efficiently solves these tasks by incorporating multiple procedures consisting of data resampling and remodelling. In our case, this eventually allows to acquire a single, simple linear model, that reapplied to patients’ metabolic data yields a value, that might be interpreted as a ‘surrogate metabolite’, being a linear combination of set of selected metabolites, which might be used for discrimination of heart failure cases, after applying a single cut-off. To our knowledge, this is the first study that implemented untargeted metabolomics analysis combined with ML algorithms in order to select metabolites creating the top performing diagnostic model for HFrEF. Recently, Verdonschot *et al*.^18^ have shown that the NT pro-BNP-based determination of the dilated cardiomyopathy (DCM) severity might be complemented by the combination of metabolites. In contrast to our study, authors have applied targeted metabolomics approach which might limit objective analysis of changes occurring in the blood/urine metabolites’ profile. The use of non-fasting blood samples, significant differences in diabetes prevalence within the studied groups, lack of chronic inflammatory disease exclusion could potentially influence the disease metabolic phenotype. Nevertheless, in spite of these study limitations the result of the analysis suggest some clinical utility of the combination of metabolomics and ML methods. In our study an untargeted metabolomics analysis was followed by computational modelling which enabled hypothesis free selection of a panel of metabolites which seemed to have non-inferior diagnostic value (based on accuracy) to BNP in chronic HFrEF. Additionally, the results of the likelihood-ratio test performed for GLMs suggested that prediction of the outcome with model utilizing BNP and eight metabolites presents significantly higher goodness-of-fit when compared with BNP alone. When we based our conclusions on data resampling, our fit slightly outperformed the BNP alone, however for such a small set, this clearly remains inconclusive.

According to the previous research, all of the selected and identified metabolites have already been described in various cardiovascular diseases (CVD) including heart failure. However, apart from DHA (possible adjunctive therapy in optimally treated patients with symptomatic HFrEF) and uric acid (useful marker of adverse prognosis in HFrEF patients), clinical significance of changes in residual components of a panel in HFrEF remains unknown. In our study HFrEF patients presented significantly higher serum UA level in comparison with the control group. According to the literature, the incidence of hyperuricemia is high in HFrEF and occurs in 50–55% of patients. An elevation in uric acid level is considered to be a result of increased production via higher xanthine oxidase activity or decreased elimination via kidneys. In our study, both groups (controls and HFrEF) were fitted in terms of estimated glomerular filtration rate (eGFR) and patients with severe chronic kidney disease were excluded, reducing the role of UA excretory disorders. It is still unclear why UA itself has a negative impact on prognosis in HFrEF^19,20^ as randomized trials with agents blocking this pathway failed to provide clinical benefit^21^. Hypothesis free selection of UA as a component of a top performing diagnostic panel confirms the need for further research and continuation of a discussion on xanthine oxidase metabolic pathway (including serum uric acid - sUA) role in HFrEF pathophysiology. Lipid disorders have also been recognized as a strong risk factor for CVD. Nevertheless, the vast majority of previous research regarding dyslipidemia in chronic HFrEF concerned solely alteration in cholesterol metabolism. As a result of a growing interest in the matter of metabolomics and lipidomics within the last years, it was possible to detect a substantial dysregulation in metabolism of other lipid fractions, including phospholipids^9,14,16^. This suggests more complex lipid metabolism abnormalities in HFrEF. In this study, serum intensities of all LPCs, likewise cholesterol level, were significantly lower in the HFrEF group. Simultaneously, higher percentage of statin use was observed in HFrEF patients. According to the results of additional analysis, it seems that statin therapy was likely to partialy impact the association between LPCs (e.i. LPC 18:2sn1) and HFrEF. Therefore, the differences between both groups (HFrEF and controls) regarding statin therapy might be considered as a possible study limitation. Although, an assessment of each particular metabolite is a kind of simplification as panel compounds should be considered together as a “surrogate metabolite”. In our previous study it has been shown that the greater serum PLs deficit, the worse clinical condition of HF patient (including more severe metabolic dysregulation, impaired renal function and decreased exercise capacity)^9^. Despite the fact that an alteration in PLs metabolism is considered to be related to plethora of processes associated with HFrEF (e.g. immune response, impaired energy metabolism, altered choline metabolism with a possible role of gut microbiota), the exact metabolic mechanism responsible for changes in PLs in HFrEF remains unknown. The dysregulation in phospholipids including phosphatidylcholines (PCs) metabolism has already been observed in various non-related diseases^9,22–25^. Lindahl *et al*.^26^ suggested that alteration in LPCs concentration may be an indicative of disease in general rather than a disease specific metabolite marker. In HFrEF which is a multisystemic syndrome, changes observed in the LPCs serum intensities considered as a part of the whole metabolites’ profile seem not to be a limitation but an advantage. The presence of LPCs in the diagnostic panel and their correlations with serum cholesterol level points out an importance of dysregulation in various lipid classes in HFrEF. Bile acids (BAs), likewise LPCs and UA, have been described as factors implicated in various cardiac pathologies. Mayerhofer *et al*.^27^ have demonstrated that the ratio of primary to secondary BAs has been reduced in chronic heart failure patients. Nevertheless, the association between this pattern of BAs composition and reduced overall survival has been seen solely in univariate analysis. In our study, serum intensity of one secondary BA (deoxycholic acid – DCA) was lower in HFrEF group. Deoxycholic acid is known as one of the two most common secondary bile acids that are synthesized solely by the microbial flora of a small intestine. An impairment in intestinal function and gut microbiome in HF has been intensively studied within the last years^28,29^. Reduced intestinal blood flow with further bowel wall oedema has been thought to be responsible for altered intestinal barrier function leading to the passage of bacterial products into the systemic blood circulation. Another component of a diagnostic panel with known anti-inflammatory, anti-arrhythmic and beneficial effects on the endothelial function was docosahexaenoic acid (DHA) classified as omega-3 polyunsaturated fatty acid (PUFA)^30,31^. Mozaffarian *et al*.^32^ have indicated that circulating omega-3 PUFAs are associated with lower incidence of chronic heart failure. In our study serum intensity of DHA was significantly lower than in the control group. Current European Society of Cardiology (ESC) guidelines for the diagnosis and treatment of acute and chronic heart failure has recommended that n-3 PUFA preparations containing >850 mg/g of eicosapentaenoic acid (EPA) and DHA may be considered as an adjunctive therapy in optimally treated patients with symptomatic HFrEF^1^.

Endothelial dysfunction, oxidative stress, systemic inflammatory activation, impaired energy metabolism, altered choline metabolism, apoptosis, intestinal dysbiosis – all of those have been thought to be implicated in HFrEF pathophysiology. As cited above, every metabolite included in the diagnostic panel has already been described to be involved in an activation of aforementioned processes. Moreover their correlations with clinical, biochemical (BNP, renal function), echocardiographic (left ventricular systolic function) and functional parameters (the distance of 6MWT, duration of exercise on a treadmill, peak VO2, VE/VCO2 slope) confirm possible clinical significance of the diagnostic panel components. Therefore, based on the results of this and previous studies, metabolomics seems to be a powerful tool in HFrEF especially when combined with ML algorithms in the detection of the top performing HFrEF diagnostic panel. Presence of additional unidentified metabolite in the panel only confirms great possibilities that the combination of those two unrestricted methods offers in the aspect of broadening the knowledge on HFrEF pathophysiology. As compared to the strategy of a single metabolite, complementarity of a panel compounds gives an opportunity to get a more complete picture of the metabolic changes taking place in the course of HFrEF and as a result may increase accuracy of the diagnostic panel.

### Study limitations

Despite the study was carefully planned we are aware that there are several potential limitations. First, number of enrolled patients is relatively small. Nevertheless, our purpose was to select the most homologous HFrEF group using strict exclusion criteria especially in terms of concomitant diseases. Second, there is no information about patients’ diet, PUFAs supplementation or HFrEF duration. Third, there are differences in pharmacotherapy between study and control group. Larger studies with prospectively followed-up groups are needed for clinical validation of the diagnostic panel.

## Conclusions

In the present study we demonstrated that the combination of two innovative methods: an untargeted metabolomics and ML algorithms can be a promising tool for the diagnostic workup in HFrEF. The combination of metabolites may provide comparable diagnostic value to BNP. Due to the complementarity of the panel components, changes in the serum intensities of particular metabolites interpreted together as a “surrogate metabolite” might be more specific to the HFrEF. Large scale, multi-center studies using validated targeted methods are crucial to confirm clinical utility of proposed markers.

## Supplementary information

Suplementary information.
