# Supplementary material for: Machine-learning facilitates selection of a novel diagnostic panel of metabolites for the detection of heart failure
Source: Sci Rep. 2020 Jan 10;10:130. doi: 10.1038/s41598-019-56889-8 (PMC6954181; doi:10.1038/s41598-019-56889-8)
Supplement: Supplementary file 1 — Suplementary information. [file 41598_2019_56889_MOESM1_ESM.docx]

**Machine-learning facilitates selection of a novel diagnostic panel of metabolites for the detection of heart failure**

M. Marcinkiewicz-Siemion^1^, M. Kaminski^1^, M. Ciborowski^2^, K. Ptaszynska-Kopczynska^1^, A. Szpakowicz^1^, A. Lisowska^1^, M. Jasiewicz^1^, E. Tarasiuk^1^, A. Kretowski^2^, B. Sobkowicz^1^, K.A. Kaminski^1,3^*

^1^ Medical University of Bialystok, Cardiology Department, M. Sklodowskiej-Curie 24A, 15-276 Bialystok, Poland; telephone number +48858318656, fax number +48858318604

^2^ Medical University of Bialystok, Clinical Research Centre, M. Sklodowskiej-Curie 24A, 15-276 Bialystok, Poland

^3^ Medical University of Bialystok, Department of Population Medicine and Civilization Diseases Prevention, Waszyngtona 13A, 15-276 Bialystok, Poland

***Corresponding author** Karol A. Kaminski, e-mail address: fizklin@wp.plfizklin@wp.pl

**Present/permanent address:** Department of Population Medicine and Civilization Diseases Prevention, Medical University of Bialystok, Waszyngtona 13a; 15-276 Bialystok, Poland

Cardiology Department, University Hospital, M. Sklodowskiej-Curie 24A; 15-276 Bialystok, Poland; telephone number +48858318656.

**SUPPLEMENTARY TABLES**

**Supplementary Table 1.** Estimates for parameters of model containing both BNP and metabolite. Likelihood ratio test, when contrasted with model containing BNP as a sole predictor: p=0.001.

| **Model Term** | **Coef. Estimate** | **Coef. Std. Error** | **P-Value** |
| --- | --- | --- | --- |
| **(Intercept)** | 0.214 | 0.98 | 0.827 |
| **BNP** | 0.017 | 0.012 | 0.135 |
| **LPC 18:2sn2** | -7.414 | 4.172 | 0.076 |
| **UA** | 0.353 | 0.465 | 0.448 |
| **LPC 18:2sn1** | 0.494 | 1.217 | 0.684 |
| **UM** | -0.815 | 1.007 | 0.418 |
| **LPC 18:2sn2** | 6.899 | 4.136 | 0.095 |
| **LPC 20:1** | -1.272 | 0.719 | 0.077 |
| **DA** | -0.471 | 0.356 | 0.186 |
| **DHA** | -0.666 | 0.426 | 0.118 |

LPC – lysophosphatidylcholine; UA – uric acid; UM – unknown metabolite; DA – deoxycholic acid; DHA – docosahexaenoic acid.

**Supplementary Table 2.** Multiple linear regression model assessing potential effect of HFrEF ischaemic etiology on metabolite levels. The following table contains p-values for model coefficients.

| **Compound** | **IHD** | **HFrEF** |
| --- | --- | --- |
| **UA** | 0.191 | **0.002** |
| **DHA** | 0.600 | **0.004** |
| **DA** | 0.084 | **0.002** |
| **LPC 18:2sn2** | **0.035** | **0.003** |
| **LPC 18:2sn1** | **0.012** | **0.040** |
| **LPC 18:2sn2** | **0.037** | **0.001** |
| **LPC 20:1** | 0.391 | **0.002** |
| **UM** | 0.085 | **0.010** |

HFrEF – heart failure with reduced ejection fraction; IHD – ischaemic heart disease as HFrEF etiology; UA – uric acid; LPC – lysophosphatidylcholine; UM – unknown metabolite; DA – deoxycholic acid; DHA – docosahexaenoic acid.

**Supplementary Table 3.** Multiple linear regression model assessing potential effect of statin therapy on metabolite levels. The following table contains p-values for model coefficients.

| **Compound** | **STATIN** | **HFrEF** |
| --- | --- | --- |
| **UA** | 0.458 | **0.005** |
| **LPC 18:2sn2** | **0.035** | **0.005** |
| **LPC 18:2sn1** | 0.113 | 0.082 |
| **UM** | 0.053 | **0.041** |
| **LPC 18:2sn2** | **0.026** | **0.016** |
| **LPC 20:1** | 0.310 | **0.005** |
| **DA** | 0.269 | **0.006** |
| **DHA** | 0.678 | **0.012** |

HFrEF – heart failure with reduced ejection fraction; UA – uric acid; LPC – lysophosphatidylcholine; UM – unknown metabolite; DA – deoxycholic acid; DHA – docosahexaenoic acid.

**Supplementary Table 4.** Multiple linear regression model assessing potential effect of ACEI therapy on metabolite levels. The following table contains p-values for model coefficients.

| **Compound** | **ACEI** | **HFrEF** |
| --- | --- | --- |
| **UA** | 0.073 | 0.052 |
| **LPC 18:2sn2** | 0.931 | **0.002** |
| **LPC 18:2sn1** | 0.815 | 0.060 |
| **UM** | 0.760 | **0.013** |
| **LPC 18:2sn2** | 0.774 | **0.009** |
| **LPC 20:1** | 0.806 | **0.003** |
| **DA** | 0.737 | **0.009** |
| **DHA** | 0.345 | **0.004** |

HFrEF – heart failure with reduced ejection fraction; ACEI - angiotensin-converting-enzyme inhibitor; UA – uric acid; LPC – lysophosphatidylcholine; UM – unknown metabolite; DA – deoxycholic acid; DHA – docosahexaenoic acid.
